# Kinin B_1 _receptors contributes to acute pain following minor surgery in humans

**DOI:** 10.1186/1744-8069-6-12

**Published:** 2010-02-13

**Authors:** May Hamza, Xiao-Min Wang, Albert Adam, Jaime S Brahim, Janet S Rowan, Gilberto N Carmona, Raymond A Dionne

**Affiliations:** 1 NINR/NIH, (10 Center drive), Bethesda, MD (20892), USA; 2 NIDCR/NIH, (10 Center drive), Bethesda, MD (20892), USA; 3 Department of Nursing, Magnuson Clinical Research Center, NIH, Bethesda, MD (20892), USA; 4 Faculty of Pharmacy, Université de Montréal, Québec (H3C 3J7), Canada; 5 Dept of Pharmacology, Faculty of Medicine, Ain Shams University, (Abbassia), Cairo (11566), Egypt

## Abstract

**Background:**

Kinins play an important role in regulation of pain and hyperalgesia after tissue injury and inflammation by activating two types of G-protein-coupled receptors, the kinin B_1 _and B_2 _receptors. It is generally accepted that the B_2 _receptor is constitutively expressed, whereas the B_1 _receptor is induced in response to inflammation. However, little is known about the regulatory effects of kinin receptors on the onset of acute inflammation and inflammatory pain in humans. The present study investigated the changes in gene expression of kinin receptors and the levels of their endogenous ligands at an early time point following tissue injury and their relation to clinical pain, as well as the effect of COX-inhibition on their expression levels.

**Results:**

Tissue injury resulted in a significant up-regulation in the gene expression of B_1 _and B_2 _receptors at 3 hours post-surgery, the onset of acute inflammatory pain. Interestingly, the up-regulation in the gene expression of B_1 _and B_2 _receptors was positively correlated to pain intensity only after ketorolac treatment, signifying an interaction between prostaglandins and kinins in the inflammatory pain process. Further, the gene expression of both B_1 _and B_2 _receptors were correlated. Following tissue injury, B_1 _ligands des-Arg^9^-BK and des-Arg^10^-KD were significantly lower at the third hour compared to the first 2 hours in both the placebo and the ketorolac treatment groups but did not differ significantly between groups. Tissue injury also resulted in the down-regulation of TRPV1 gene expression at 3 hours post-surgery with no significant effect by ketorolac treatment. Interestingly, the change in gene expression of TRPV1 was correlated to the change in gene expression of B_1 _receptor but not B_2 _receptor.

**Conclusions:**

These results provide evidence at the transcriptional level in a clinical model of tissue injury that up-regulation of kinin receptors are involved in the development of the early phase of inflammation and inflammatory pain. The up-regulation of B_1 _receptors may contribute to acute inflammatory pain through TRPV1 activation.

## Background

Tissue injury results in the liberation of various pain and inflammatory mediators; we have reported earlier in the oral surgery model of acute inflammatory pain the production or up-regulation of a number of prostanoids, cytokines and chemokines [1-4] following tissue injury. Hargreaves and colleagues have also shown an increase in bradykinin (BK) concentration following third molar tooth extraction [5]. Further, they showed that the NSAID flurbiprofen prevented this increase in BK levels.

Bradykinin-related peptides, collectively known as kinins, are proinflammatory mediators that mediate vascular responses and pain following tissue injury. Kinins bind to two types of G protein-coupled receptors, the B_1 _and B_2 _receptors, both of which have been cloned [6,7]. B_1 _receptors are activated by the endogenous kinins lacking the carboxy-terminal Arg residue, namely des-Arg^9^-BK and Lys-des-Arg^9^-BK, also known as des-Arg^10^-KD. B_2 _receptors are activated by the full sequence of the endogenous kinins BK, and Lys-BK, also known as kallidin (KD) [8]. B_2 _receptors are constitutively expressed but undergo extensive desensitization by their agonists. They are widely distributed and mediate most of the biological actions of BK. On the other hand, B_1 _receptors are induced during the inflammatory processes or at least strongly regulated, except in the spinal cord, where they are constitutively expressed in both rat and man [9]. Further, B_1 _receptors are only subjected to limited desensitization, which make them a better target for analgesics [10,11]. In experimental studies, the expression of B_1 _receptors has been reported to occur in response to the B_1 _ligand Lys-des-Arg^9^-BK [12] and inflammatory cytokines such as IL-1β and TNF-α [13-15]. Regulation of B_1 _expression by B_2 _receptor through activation of NFκB and MAP kinases has also been observed [12,15]. However, to our knowledge this has not been shown in man.

Kinin receptors are expressed in neuronal tissues, are upregulated in response to painful stimuli and their antagonists produce an antinociceptive effect in different pain models [16,17]. Furthermore, B_1 _receptor knockout mice are refractory to chemical and thermal nociceptive stimuli [18]. Transient receptor potential vanilloid 1 (TRPV1) is suggested to mediate ionic mechanisms coupling BK receptors to the excitation and sensitization of nociceptors [19].

The interaction between prostaglandins (PG) and BK in the process of inflammatory pain is well established [20,21] as BK induces prostaglandin release in various tissues [20,22,23]. It is suggested that BK-induced sensitization is in part secondary to prostaglandin synthesis, since NSAIDs inhibit BK-mediated sensitization of heat responses, while prostaglandin E_2_/I_2 _reverse this inhibition [24,25].

The aim of the present study was to investigate the role of kinin receptors in acute inflammatory pain in humans by assessing the gene expression of B_1 _and B_2 _receptors following oral surgery and its correlation to self-reported pain intensity, as well as to evaluate the levels of their immunoreactive ligands at the site of tissue injury using the microdialysis technique. The interaction between the kinin system and COX-PG pathway was also studied.

## Results

### 1. Effect of tissue injury and ketorolac treatment on BK and des-Arg^9^-BK

**Figure 1:**
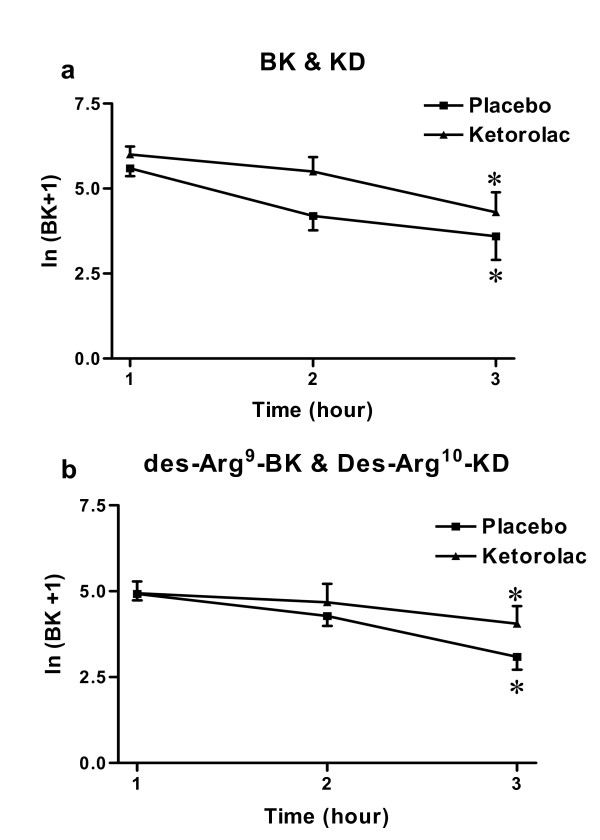
**The levels of immunoreactive kinins were significantly lower at the third hour compared to the first 2 hours**. There was no significant difference between the placebo and ketorolac treatment groups. Fig. (1a) shows the levels of B_2 _receptor ligands (BK and KD) and Fig. (1b) shows the levels of B_1 _receptor ligands (des-Arg^9^-BK and des-Arg^10^-KD). Concentrations of immunoreactive kinins (pg/ml) were transformed into Ln (X+1), which is shown here. For details, please refer to the methods section. Figures show the levels measured at the left side. The right side showed similar results. Data are presented as mean ± SEM; *indicated p < 0.05, 3 way ANOVA.

Both BK and des-Arg^9^-BK levels in microdialysate were detectable at all time points and decreased gradually over the 3 h collection period with the third hour being significantly lower than the first and second hours in both the placebo and ketorolac treatment groups (p < 0.05). However, there was no significant difference between both treatment groups and Fig. 1 (a&b) shows the levels measured at the left side. The right side showed the same results (data not shown).

HPLC analysis performed on microdialysate samples collected at the site of tissue injury from both placebo and ketorolac treatment groups, showed that kinins detected by chemiluminescent enzyme immunoassays were mainly detected at the retention time corresponding to the reference peptides des-Arg^9^-BK (18.6 min), BK (24.1 min), des-Arg^10^-KD (24.7 min) and KD (29.8 min).

### 2. Effect of tissue injury and ketorolac treatment on *BDKRB1 *and *BDKRB2 *gene expression at the site of tissue injury

Following the identification of four different immunoreactive kinins and their increased levels following tissue injury, it becomes of interest to test for the expression of kinin receptors in the same model. Both *BDKRB1 *and *BDKRB2 *were significantly upregulated 3 hours following tissue injury in both the placebo and ketorolac treatment group (Table 1). *BDKRB1 *was 2.5-fold upregulated in the placebo treated group (p < 0.0001; paired t-test; n = 15) and 2.4-fold upregulated in the ketorolac treatment group (p < 0.0001; paired t-test; n = 15). Similarly, *BDKRB2 *was 2-fold upregulated in the placebo treated group (p < 0.0001; paired t-test; n = 15) and 1.9-fold upregulated in the ketorolac treatment group (p < 0.0001; paired t-test; n = 15). Ketorolac treatment did not have a significant effect on the gene expression of either *BDKRB1 *(p = 0.71; unpaired t-test) or *BDKRB2 *(p = 0.73; unpaired t-test).

**Table 1:** Gene expression of kinin receptors, TRPV1 and NFκB:

	Placebo			Ketorolac		
	
	Pre-surgery (Average ΔCt)	Post-surgery (Average ΔCt)	Fold change* (P value)	Pre-surgery (Average ΔCt)	Post-surgery (Average ΔCt)	Fold change* (P value)
**B_1_receptor** *(BDKRB1)*	17.4 ± 3.6	16.2 ± 3.6	2.5 ± 0.9 (p < 0.0001)	18.6 ± 3.4	17.4 ± 3.5	2.4 ± 0.8 (p < 0.0001)

**B_2_receptor** *(BDKRB2)*	14.8 ± 3.0	13.8 ± 2.9	2.0 ± 0.6 (p < 0.0001)	15.6 ± 2.7	14.7 ± 2.7	1.9 ± 0.5 (p < 0.0001)

**NFκB**	17.3 ± 0.6	17.0 ± 0.4	1.3 ± 1.2 (p = 0.027)	17.3 ± 0.5	17.0 ± 0.7	1.4 ± 0.8 (p = 0.042)

**TRPV1**	18.8 ± 2.9	19.8 ± 2.7	-2.2 ± 0.9 (p < 0.0001)	19.5 ± 2.7	20.7 ± 2.9	-2.6 ± 1.1 (p < 0.0001)

Data are presented as mean ± S.D.; n = 13-15 per group.

*Fold change is calculated as 2^-ΔΔCt^, Average ΔC_t _is the normalized cycle threshold in reference to the endogenous control (18S rRNA). Data were analyzed using paired t test.

### 3. Correlation between pain intensity and the gene expression of BK receptors

The contribution of kinins to pain is well established. Further, the correlation between BK and KD and change in pain intensity was recently reported in man [26]. We assessed here whether the gene expression of kinin receptors was also correlated to pain intensity.

**Figure 2:**
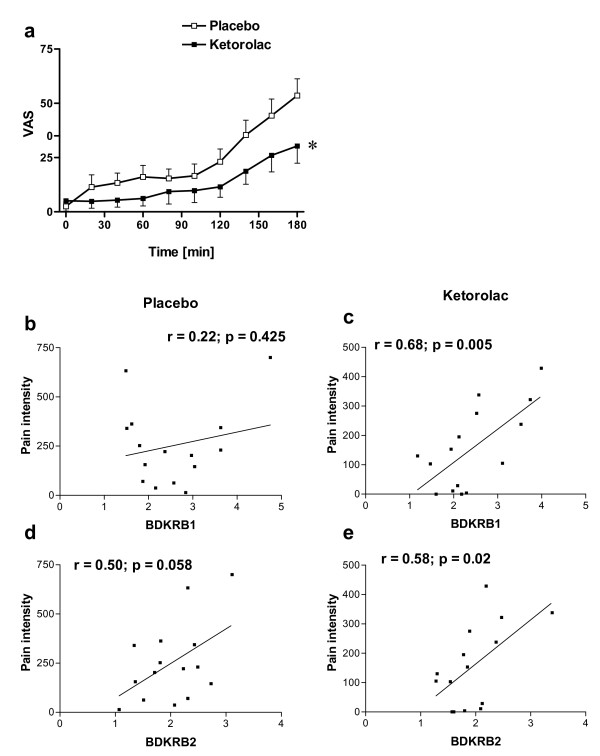
**(a) Pain intensity assessed by 100 mm VAS was lower in the keterolac treatment group (p = 0.023, two way ANOVA); Data are presented as mean ± SEM**. (b-e) The change in gene expression level (RQ) of both B1 and B2 receptors was correlated to pain intensity (VAS) in the keterolac treatment group but not in the placebo treatment group. The association between the gene expression and pain scale was examined using Pearson correlation. The y-axis represents the sum of the pain intensity over the first 3 hours post-surgery. The x-axis represents the relative changes in gene expression (RQ) from qRT-PCR.

Pain intensity has increased throughout the three hours observation period (time: *p *< 0.001), and as expected ketorolac treatment induced a significant analgesic effect (p = 0.023; two-way repeated measure ANOVA) over the three hours observation period (Fig. 2a).

The correlation between the sum of pain intensity scores as measured by VAS and the up-regulation of gene expression of *BDKRB1 *and *BDKRB2 *following tissue injury was examined using Pearson's correlation coefficients (Fig. 2 b-e). Both *BDKRB1 *(r = 0.68; p = 0.005; n = 15) and *BDKRB2 *(r = 0.58; p = 0.02; n = 15) were positively correlated to pain intensity in the ketorolac treatment group. On the other hand, *BDKRB1 *(r = 0.22; p = 0.425; n = 15) and *BDKRB2 *(r = 0.50; p = 0.058; n = 15) did not significantly correlate to pain intensity in the placebo treatment group.

### 4. Effect of tissue injury and ketorolac treatment on TRPV1 and NFκB gene expression at the site of tissue injury

To investigate the mechanism of B_1 _receptor up-regulation and the contribution of TRPV1 receptor to the algesic mechanisms of both kinin receptors, we further evaluated the gene expression of both TRPV1 and NFκB in the same biopsies and examined the correlation among the changes in their gene expression levels.

NFκB was slightly, though significantly, up-regulated following tissue injury in both the placebo treated group (1.3-fold, p = 0.027; paired t-test; n = 15) and the ketorolac treatment group (1.4-fold, p = 0.042; paired t-test; n = 13; Table 1). There was no significant difference between the two treatment groups (p = 0.56; unpaired t-test).

On the other hand, TRPV1 was significantly down regulated following tissue injury in both the placebo treated group (2.2-fold, p < 0.0001; paired t-test; n = 15) and the ketorolac treatment group (2.6-fold, p < 0.0001; paired t-test; n = 15). However, there was no significant difference between the two treatment groups (p = 0.33; unpaired t-test).

### 5. Correlation between the gene expression of *BDKRB1*, *BDKRB2*, TRPV1 and NFκB

**Figure 3:**
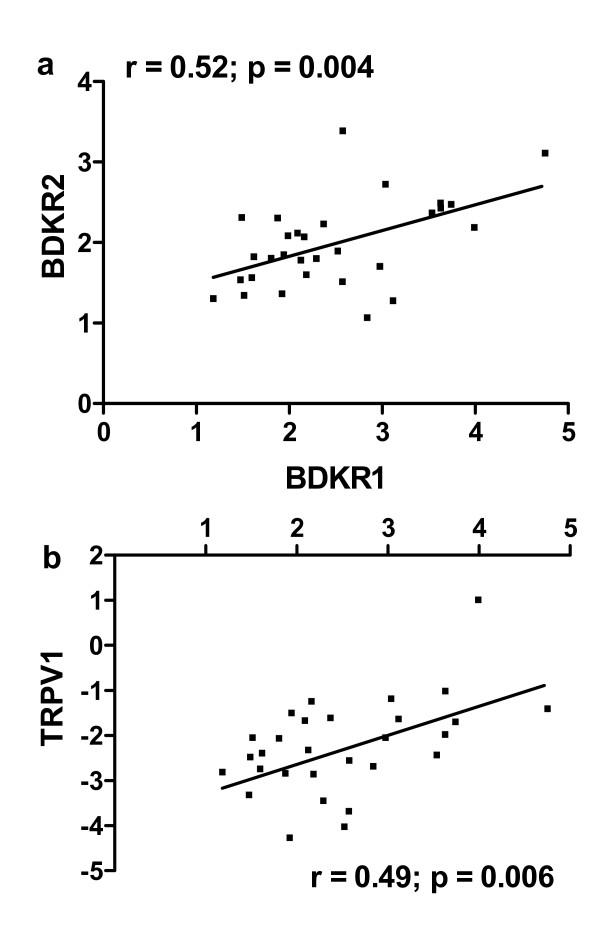
**(a) The relative changes in gene expression (RQ) from qRT-PCR of B1 and B2 receptors were correlated**. (b) Similarly, the relative change in gene expression of TRPV1 was correlated to that of B1 receptor. The relative change in gene expression is calculated as 2^-ΔΔCt ^to show the fold increase. In case of TRPV1, the downregulation is shown as the negative fold change. The association was examined using Pearson correlation at 3 hours post-surgery.

Since there was no significant difference between the two treatment groups (placebo and ketorolac) in the gene expression of either of the four genes studied, the two treatment groups were pooled to calculate the correlation coefficient for both receptors [4]. The gene expression of *BDKRB1 *and *BDKRB2 *were significantly correlated (r = 0.52; p = 0.004; n = 30; Fig. 3a). Further, the gene expression of TRPV1 was significantly correlated to *BDKRB1 *(r = 0.49; p = 0.006; n = 30; Fig. 3b) but not *BDKRB2 *(r = 0.33; p = 0.077; n = 30; data not shown). On the other hand, the gene expression of NFκB was not correlated to either *BDKRB1 *(r = -0.106; p = 0.61; n = 26) or *BDKRB2 *(r = 0.19; p = 0.35).

## Discussion

Kinin levels are very difficult to measure accurately in biological samples, particularly in blood, due to the presence of metabolic enzymes that have the capacity to synthesize or metabolize kinins in vitro. Further, kinins act locally close to their site of formation before they are rapidly hydrolyzed [27]. The microdialysis technique overcomes these difficulties by collecting the kinins at the site of inflammation and excluding the large molecular weight enzymes that may change kinin levels.

In the present study, and in accordance with previous studies [5,26], we show an elevation in the tissue levels of BK and KD following tissue injury. While the chemiluminoenzyme immunoassay does not distinguish between the two peptides, the use of immuno-HPLC approach showed clearly that both peptides were present in the microdialysis samples. Since microdialysis sampling in this study started at the completion of third molar extraction (approximately 30 minutes duration), and the intervals of sampling was relatively long (1 hour) to allow for sufficient volume of each sample, the rise of immunoreactive peptide concentration was not detected, but rather the fall of its concentration 3 hours following surgery was detected. This is expected since the concentration of both kinins are rapidly elevated within 15 min following injury [26].

Further, we show for the first time in an in-vivo human study, a similar pattern for B_1 _receptor ligands (des-Arg^9^-BK, des-Arg^10^-KD), being elevated following tissue injury before their levels decrease at the latter time points. The immuno-HPLC approach provides evidence for the presence of des-Arg^10^-KD, which is the most potent known human B_1 _receptor agonist [28]. Most of the earlier studies of acute inflammatory pain focused on BK and KD due to the impression that the B_1 _receptors are only expressed in chronic inflammation [29]. However, we show here that the B_1 _receptor is up-regulated as early as 3 hours following tissue injury in humans. Further, pre-surgery biopsies showed expression of both B_1 _and B_2 _mRNA before any tissue injury. This early expression of B_1 _receptor [30] or even its constitutive expression [31] has been shown earlier in some experimental studies. The importance of B_1 _receptor lies in the role that it plays in maintaining inflammation in chronic pain states, in contrast to the B_2 _receptor that is quickly desensitized. Several mechanisms have been suggested to induce B_1 _receptor up-regulation in response to inflammation. First, it may be cross up-regulated by activation of B_2 _receptors [12]. The correlation we show here between the gene expression of B_2 _and B_1 _receptor supports this mechanism in the present clinical model of tissue injury. Second, our earlier study showed marked up-regulation of IL-1β [1] and TNF-α [2] following surgery, and TNF-α was densely expressed on infiltrated inflammatory cells, particularly, on macrophages. Given both TNF-α and IL-1β are known to upregulate B_1 _receptor both in-vitro [32] and in-vivo [15], we hypothesize that the up-regulation of both IL-1β and TNF-α may contribute to the up-regulation of B_1 _shown in this study. Finally, our recent study demonstrates that an inflammatory cascade mediated by IL-6, IL-8, CCL2, CXCL1 and CXCL2 contributes to the development of acute inflammation and inflammatory pain [4]. This cascade may also contribute to the B_1 _receptor up-regulation since neutrophil influx has been suggested to contribute to the up-regulation of B_1 _receptors [15].

The activation of the transcription factor NFκB is a critical step in up-regulation of B_1 _receptors that are induced not only by IL-1β and TNF-α [15,33], but also by its own agonists [34] or by activation of B_2 _receptors [35]. The small change in NFκB gene expression suggests that this may be mediated primarily at the protein level (i.e. the activation of NFκB) and the role of NFκB gene expression is relatively small at the early stage of this clinical model of tissue injury. However, to confirm the role of activation of NFκB in this model further analysis at the protein level is warranted. Due to the limited size of the biopsy, we were unable to conduct the study at both the protein and the gene expression levels.

**Figure 4:**
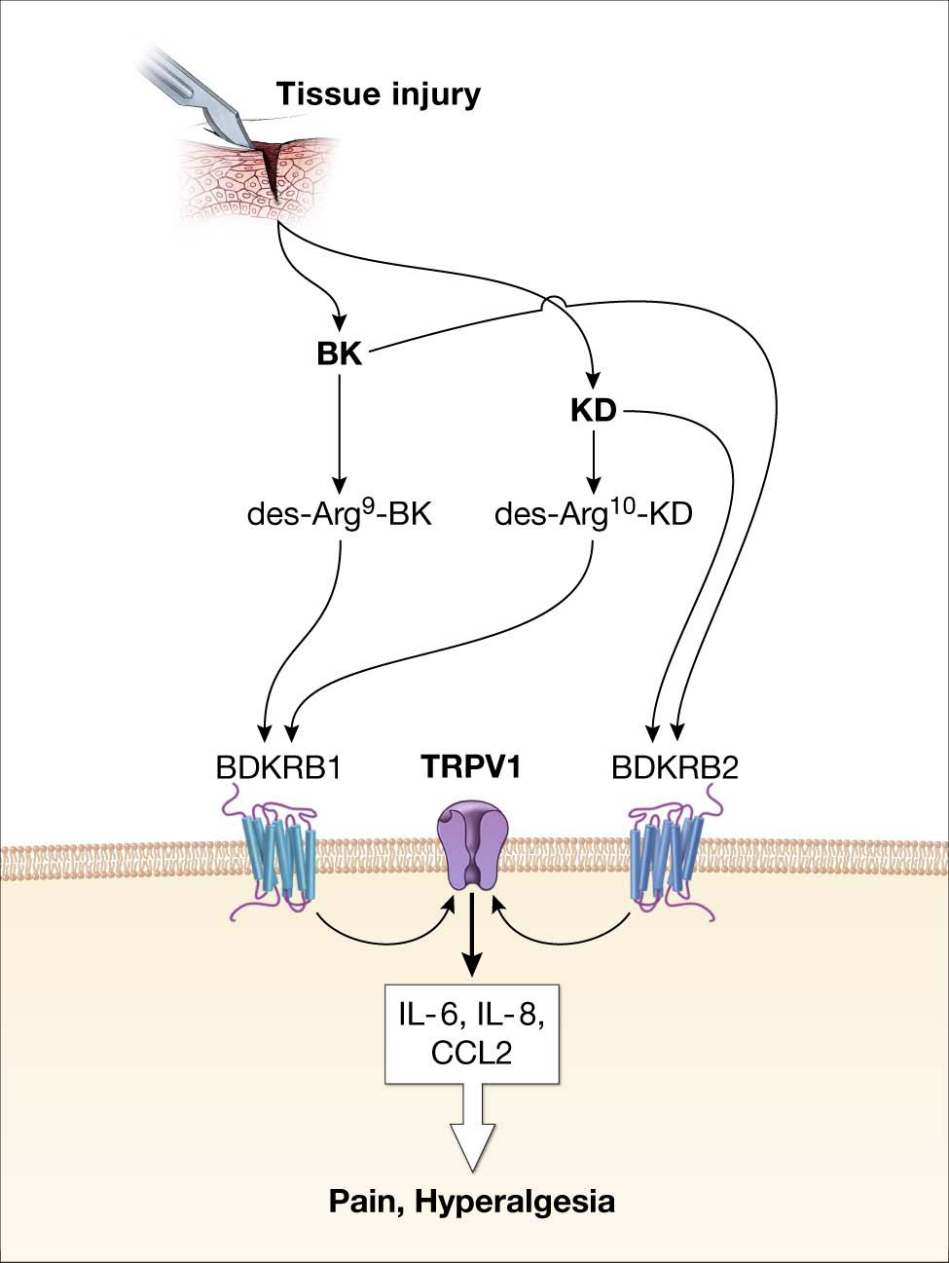
**Tissue injury results in the production of kinins and cytokines**. Kinins activate both B_1 _and B_2 _receptors on nerve ending and on other cells (possibly, gingival fibroblasts, epithelial cells or inflammatory cells). The activation of both B_1 _and B_2 _receptors leads via different signaling pathways to the production of IL-6, IL-8 and CLL2, all of which can activate their corresponding receptors on the sensory nerve endings. Activation of TRPV1 is thought to mediate kinins effects on sensory neurons and might possibly mediate their effect on other cells to produce cytokines.

The role of kinin receptors and their endogenous ligands in inflammation and pain is well recognized (Fig. 4). Not only does activation of B_2 _receptors on primary sensory neurons lead, via second messenger pathways, to the activation of polymodal nociceptors and hyperalgesia [29], but bradykinin can also sensitize nociceptors following the release of prostaglandins [22,25]. This might explain the correlation between pain intensity and B_2 _receptors expression in the ketorolac treatment group but not in the placebo treatment group. The presence of prostaglandins affects pain intensity, therefore, confounding the correlation between B_2 _receptor and pain intensity shown in the absence of prostaglandins. Prostanoids released in response to tissue injury [1] would contribute to the inflammatory pain response by activating their receptors on nerve endings as well as by interacting with mediators released to sensitize nociceptors. The similar correlation between B_1 _expression and pain intensity points to the participation of B_1 _receptor in pain processes at this early time point (3 hours) in contrast to earlier reports [16] confining the contribution of B_1 _receptors to nociception to later time points. While the present data shows only the gene expression of kinin receptors due to limited biopsy size, a correlation between B_1 _gene expression level and functional up-regulation of the receptor has been reported earlier in LPS treated rats [15]. Further, the correlation between pain intensity and gene expression level reported here, argues in favor of a functional relationship between tissue injury and increased BK receptor activation leading to an increase in pain.

Kinin receptors are known to be expressed in different tissues besides primary sensory neurons. Immunohistochemical studies showed immunoreactivity for both B_1 _and B_2 _receptors in epithelial cells, submucosal glands, fibroblast, vascular smooth muscle, vascular endothelial cells, and macrophages of human nasal turbinates [36]. Further, B_1 _and B_2 _receptor genes are expressed in gingival fibroblasts [37]. Inflammatory cells such as mast cells also, shows expression of B_1 _and B_2 _receptor [38]. Thus, cellular localization of B_1 _and B_2 _receptor in the present study could be gingival fibroblasts, epithelial cells in oral mucosa or the inflammatory cellular infiltrate. However, since we did not carry out immunohistochemical studies, we cannot be certain of the cell types of the kinin receptors in oral mucosa, as qRT-PCR cannot distinguish the cellular origin of mRNA expressed.

In gingival fibroblasts, BK up-regulates IL-1β and TNFα-stimulated IL-6 [39] and IL-8 [40] production. Several other studies showed an induction of IL-6, IL-8 and CCL2 via activation of B_1 _or B_2 _receptors in a variety of cells [41-47]. Protein kinase C (PKC) signaling pathway seems to be responsible for BK induced IL-8 production in gingival fibroblasts [40], and PI-PLC and NF-κB signaling pathways are thought to be responsible for BK increased IL-6 production, in synovial fibroblasts [47]. As mentioned earlier, we have recently shown a correlation between the gene expression of IL-6, IL-8 and CCL2 and pain intensity, in the oral surgery model [4]. Therefore, it is possible that kinins besides activating B_1 _and B_2 _receptors on sensory nerve terminals, also activate B_1 _and B_2 _receptors on gingival fibroblasts or inflammatory cells inducing the expression of these cytokines, which contributes to the development of inflammatory pain.

TRPV1, a non-selective cation channel expressed in a group of sensory neurons [48], is considered an important target of BK signaling pathways to activate sensory neurons [19]. However, several other tissues express TRPV1 in humans, including keratinocytes [49], mast cells [50], synovial cells [51] and dental pulp fibroblasts [52]. Activation of the TRPV1 results in an increased release of IL-8 and PGE_2 _by epidermal keratinocytes [49], IL-6 [53] and IL-8 [54] by human bronchial epithelial cells and IL-6 by dental pulp fibroblast [52]. Engler et al. showed an increased gene and protein expression of IL-6 in cultured synovial fibroblasts from osteoarthritis (OA) and rheumatoid arthritis (RA) patients in response to the TRPV1 agonist capsaicin, suggesting a possible non-neuronal role for TRPV1 in OA and RA pain [51]. Therefore, it is possible that TRPV1 represent a target of BK signaling pathways both to induce cytokines by non-neuronal cells and to activate sensory neurons. It is suggested that B_2 _receptor via PKC signaling pathway activates TRPV1 in neuronal cells [55]. The activation of TRPV1 by B_1 _receptors was also suggested earlier [56,57]. This would pose a plausible explanation to the correlation between the change in gene expression of both receptors even with the up-regulation of B_1 _and down-regulation of TRPV1.

In the present study, we did not see a correlation between B_2 _receptor and TRPV1 gene expression. The absence of significant correlation between B_2 _and TRPV1 gene expression could possibly reflect the dissociation between gene expression levels and the functionality of B_2 _receptors due to the constitutive nature of B_2 _receptors and their rapid desensitization. It could also be due to the contribution of other TRP receptors to the signaling mechanism of B_2 _receptors. Recently, using TRPV1-KO mice, TRPV1-independent mechanisms were shown to contribute to BK-evoked pain responses [58], which might include TRPA1 and other ion channels [59]. The down-regulation of TRPV1 in response to tissue injury is however, an unexpected finding, given its pronociceptive role [60]. Yet, the same finding was seen following spinal cord injury in rat [61], where TRPV-1 receptor gene expression levels were decreased at the level of spinal cord injury but increased at rostral spinal cord areas. The up-regulation of TRPV1 is frequently reported at the spinal level following neuropathic pain models [61,62]). It is possible that TRPV1 would play different roles in the periphery or at the spinal level.

In conclusion, we propose here that B_1 _receptor is upregulated early following tissue injury in man, possibly in response to kinin activation, and cytokines and chemokines secretion. B_1 _receptor activation contributes to the development of acute inflammatory pain possibly via the activation of TRPV1.

## Methods

### Subjects, timeline of clinical procedures and biopsy collection

Healthy volunteers (n = 59) between 16 to 30 years old who required extraction of impacted third molars were included in the present study (Table 2). The protocol was approved by the Institutional Review Board of the National Institute of Dental and Craniofacial Research, National Institutes of Health (NIH). Written informed consent was obtained from all participants before treatment. Pregnant or lactating females or patients with the presence of clinical signs of infection or inflammation at the extraction sites were not included in the study. All subjects received intravenous midazolam (4.8 ± 0.5 mg) and mandibular nerve block with 2% lidocaine (164.6 ± 26.9 mg) with epinephrine 1:100,000 prior to surgery.

**Table 2:** Demographic data of participants:

	Microdialysis		Biopsies	
**Treatment**	**Placebo**	**Ketorolac**	**Placebo**	**Ketorolac**

**Number**	15	14	15	15

**Age**	22.3 ± 3.5	19.2 ± 2.9	19 ± 3.6	18.8 ± 2.6

**Gender M/F**	5/10	4/10	8/7	10/5

**Race**				

**White**	5	11	12	11

**African**	3	1	1	2

**Others**	7	2	2	2

Difficulty*	7.1 ± 2.1	7.1 ± 1.0	7.5 ± 0.9	7.2 ± 0.9

**Rescue Medicine**	11 (73%)	7 (50%)	8 (53%)	4 (27%)

* Extraction difficulty is the sum calculated by assigning a score of (2) for soft tissue impactions, (3) for partial bony impactions and (4) for full bony impactions. Data are presented as mean ± S.D.

For 29 patients, following satisfactory local anesthesia, two mandibular third molars were extracted and a surgical difficulty score was assigned for each tooth as follows: a score of (2) for soft tissue impactions, (3) for partial bony impactions and (4) for full bony impactions. After extraction, a microdialysis probe (CMA/20 Microdialysis Probe; CMA/Microdialysis, North Chelmsford, MA) was placed bilaterally along the buccal aspect of the mandible, beneath the mucogingival flap elevated for the surgical procedure. The probe fiber consists of a 10-mm flexible, nonmetallic, semipermeable dialysis membrane with a molecular cutoff ranging from 3000 to 20,000 Da. The probes were secured to an adjacent tooth with silk suture and the flap closed in the usual fashion using 3-0 chromic gut suture. Sterile lactated Ringer's solution was pumped at 10 μL/min and samples collected at 60-min intervals after the completion of surgery, before pain onset. Subjects randomly received either placebo or ketorolac (30 mg) intravenously 30 min before biopsy or microdialysis sampling. They remained under observation for the first 3 h after surgery to evaluate pain and adverse events and collect samples by microdialysis. During the immediate postoperative period, subjects were allowed one dose of tramadol (100 mg) as a rescue medication, if requested, after 1 h of evaluation. Patients rated their pain intensity every 20 min for the first 3 postoperative hours using a 100-mm visual analog scale. At the conclusion of the observation period, the microdialysis probes were removed. Microdialysis samples were placed on dry ice after each collection period, and stored at -70°C. For the other 30 patients, a preoperative 3 mm punch biopsy was taken from the oral mucosa overlying the impacted third molar and a second biopsy was taken from a different surgical site 3 hours post-surgery. All biopsies were immediately frozen in liquid nitrogen and stored at -70°C until ready for RNA extraction.

### Quantitative real-time PCR

Oral mucosal biopsies (*n *= 60) were used to detect gene expression using ABI Prism 7900 HT Sequence Detection System (Applied Biosystems, Foster City, CA) as described previously [2,3,63]. All reagents were purchased from Applied Biosystems and 2 μg of DNase-treated RNA was used to synthesize cDNA using random primers from the High-Capacity cDNA Archive Kit according to the manufacturer's instruction. Polymerase chain reaction was performed with cDNA template using the PCR Master Mix with AmpErase UNG. Sequence-specific primers and TaqMan MGB probes were purchased from Assays-on-Demand Gene expression product. Quantification of gene expression was performed in a 20-μL reaction (384-well plate) and each sample was run in triplicate. The housekeeping gene 18S rRNA was used as endogenous control and negative controls were processed under the same conditions without a cDNA template. Data acquisition was conducted using User Bulletin #2 software (v1.6, Applied Biosystems). The threshold cycle (Ct) of 18S rRNA was used to normalize target gene expression (ΔCt) to correct for experimental variations and average ΔCt was used for comparison of the gene expression in post-surgery tissue versus that in pre-surgery tissue. The relative change in gene expression calculated as 2^-ΔΔCt ^was used to compare different treatment groups.

### Quantification of BK and des-Arg^9^-BK

Microdialysis samples were diluted in cold absolute ethanol (1:8 v/v), incubated overnight at 4°C then centrifugated. The supernatant was evaporated to dryness and the residue of evaporation was stored at - 20°C until the quantification and characterization of immunoreactive kinins. Residues of evaporated ethanol extracts of microdialysates were resuspended in 50 mM Tris-HCl buffer pH 7.4, containing 100 mM NaCl and 0.05% Tween-20. After resuspension, B_2 _receptor ligands (BK and KD) and B_1 _receptor ligands (des-Arg^9^-BK and des-Arg^10^-KD) were quantified by competitive chemiluminescent enzyme immunoassays as previously described [64,65]. These methods have been validated and their analytical performances were reported. As described earlier, these immunoassays shows cross reactivity between BK and KD and between des-Arg^9^-BK and des-Arg^10^-KD.

Identification of immunoreactive kinins (BK, KD, des-Arg^9^-BK, des-Arg^10^-KD) was carried out using HPLC (Agilent 1100 Series system, Agilent Technologies Canada, Mississauga, ON, Canada) as described earlier [66]. Briefly, 40 microdialysis samples were pooled, and extracted with cold ethanol as described above. The residue of samples was dissolved in 200 μl of 5 mM KH_2_PO_4_, pH 3.0, 25% acetonitrile with 1% H_3_PO_4_. BK, KD, des-Arg^9^-BK, des-Arg^10^-KD were separated on a 2-sulfoethyl aspartamide column (PolySULFOETHYL A, The Nest Group Inc., Southborough, MA, USA) using a linear gradient of KCl (0-300 mM) in 5 mM KH_2_PO_4 _and 25% acetonitrile (v/v) pH 3 for 30 min. Fractions of 1 ml were collected from minutes 15 to 35, evaporated to dryness before quantification of immunoreactive BK and des-Arg^9^-BK. Retention time of each immunoreactive fraction was compared to that of standard peptides: des-Arg^9^-BK (18.6 min), BK (24.1 min), des-Arg^10^-KD (24.7 min) and KD (29.8 min).

### Statistical Analysis

All statistical analyses were conducted using SPSS (v. 16.0, SPSS, Chicago, IL). Paired t-test was used to compare the gene expression in post-surgery tissue versus that in pre-surgery tissue. The relative change in gene expression was expressed as 2^-ΔΔCt ^and was used to compare the change in gene expression in different treatment groups by unpaired t-test. Linear regression analysis was used to examine the association between the fold changes in gene expression (2^-ΔΔCt^) and the sum of patient-reported pain scores over the first 3 h post-surgery as measured by VAS. In the case of down- regulation, the negative fold change was used. The association among these gene expressed was examined by Pearson's correlation coefficients.

The effect of ketorolac treatment on VAS was assessed using repeated measures two-way ANOVA and the effect of ketorolac treatment on immunoreactive kinins was assessed using three-way ANOVA with two within subject factors (side and time) and one between subjects factor (treatment). A contrast analysis using t-tests with Bonferroni adjustment was used to measure the differences, which were statistically significant. Data did not follow normal distribution, so was transformed to ln(x+1). Results were considered significant at p < 0.05.

## Competing interests

The authors declare that they have no competing interests.

## Authors' contributions

MH participated in the laboratory experimental design, conducted qRT-PCR experiments, data analysis and drafted manuscript. XMW participated in the laboratory experimental design, conducted qRT-PCR experiments, data analysis and partially participated in the manuscript writing. AA conducted the HPLC quantification of bradykinin and des-Arg^9^-BK analysis and data collection. GNC contributed to patient biopsy collection and data collection. JSB, JSR and RAD participated in the patient enrollment, surgical procedures, patient care and biopsy collection. RAD was entirely responsible for the overall study design, overseeing data collection, analysis and interpretation as well as manuscript version. All authors have read and approved the final manuscript.
